# Circulating prolactin and *in situ* breast cancer risk in the European EPIC cohort: a case-control study

**DOI:** 10.1186/s13058-015-0563-6

**Published:** 2015-03-31

**Authors:** Kaja Tikk, Disorn Sookthai, Renée T Fortner, Theron Johnson, Sabina Rinaldi, Isabelle Romieu, Anne Tjønneland, Anja Olsen, Kim Overvad, Françoise Clavel-Chapelon, Laura Baglietto, Heiner Boeing, Antonia Trichopoulou, Pagona Lagiou, Dimitrios Trichopoulos, Giovanna Masala, Vittorio Krogh, Rosario Tumino, Fulvio Ricceri, Amalia Mattiello, Antonio Agudo, Virginia Menéndez, María-José Sánchez, Pilar Amiano, Maria-Dolores Chirlaque, Aurelio Barricarte, HBas Bueno-de-Mesquita, Evelyn M Monninkhof, N Charlotte Onland-Moret, Anne Andresson, Malin Sund, Elisabete Weiderpass, Kay-Tee Khaw, Timothy J Key, Ruth C Travis, Melissa A Merritt, Elio Riboli, Laure Dossus, Rudolf Kaaks

**Affiliations:** Division of Cancer Epidemiology, German Cancer Research Center (DKFZ), Heidelberg, Germany; Section of Nutrition and Metabolism, International Agency for Research on Cancer (IARC), Lyon, France; Danish Cancer Society Research Center, Copenhagen, Denmark; Section for Epidemiology, Department of Public Health, Aarhus University, Aarhus, Denmark; INSERM, Centre for Research in Epidemiology and Population Health [CESP], U1018, Nutrition, Hormones and Women’s Health team, F-94805 Villejuif, France; University Paris Sud, UMRS 1018, F-94805 Villejuif, France; IGR, F-94805 Villejuif, France; Cancer Epidemiology Centre, Cancer Council Victoria, 3053 Melbourne, Australia; Centre for Molecular, Environmental, Genetic, and Analytic Epidemiology, The University of Melbourne, 3010 Melbourne, Australia; Department of Epidemiology, German Institute of Human Nutrition (DIfE) Potsdam-Rehbrücke, Nuthetal, Germany; Hellenic Health Foundation, 13 Kaisareias Street, GR-115 27 Athens, Greece; Department of Hygiene, Epidemiology and Medical Statistics, University of Athens Medical School, 75 M. Asias Street, Goudi GR-115 27 Athens, Greece; Bureau of Epidemiologic Research, Academy of Athens, 23 Alexandroupoleos Street, Athens, GR-115 27 Greece; Department of Epidemiology, Harvard School of Public Health, 677 Huntington Avenue, Boston, USA; Molecular and Nutritional Epidemiology Unit, Cancer Research and Prevention Institute – ISPO, Florence, Italy; Epidemiology and Prevention Unit, Fondazione IRCCS Istituto Nazionale dei Tumori, Via Venezian 1, Milano, Italy; Cancer Registry and Histopathology Unit, “Civic - M.P.Arezzo” Hospital ASP, Ragusa, Italy; Unit of Cancer Epidemiology, AO Citta’ della Salute e della Scienza, University of Turin, Torino, Italy; Center for Cancer Prevention (CPO), Via Santena 7, 10126 Torino, Italy; Department of Clinical and Experimental Medicine, Federico II University, Naples, Italy; Unit of Nutrition, Environment and Cancer, Catalan Institute of Oncology-ICO, IDIBELL, L’Hospitalet de Llobregat, Barcelona, 08908 Spain; Public Health Directorate, Asturias, Spain; Escuela Andaluza de Salud Pública, Instituto de Investigación Biosanitario de Granada (Granada.ibs), Hospitales Universitarios de Granada/Universidad de Granada, Granada, Spain; Public Health Division of Gipuzkoa, BioDonostia Reserach Institute, San Sebastian, Spain; Department of Epidemiology, Murcia Regional Health Authority, Murcia, Spain; Navarre Public Health Institute, Pamplona, Spain; Consortium for Biomedical Research in Epidemiology and Public Health (CIBER de Epidemiología y Salud Pública (CIBERESP)), Madrid, Spain; Department for Determinants of Chronic Diseases (DCD), National Institute for Public Health and the Environment (RIVM), Bilthoven, The Netherlands; Department of Gastroenterology and Hepatology, University Medical Centre, Utrecht, The Netherlands; Department of Epidemiology and Biostatistics, The School of Public Health, Imperial College, London, UK; Department of Social & Preventive Medicine, Faculty of Medicine, University of Malaya, Kuala Lumpur, Malaysia; Department of Epidemiology, Julius Center for Health Sciences and Primary Care, University Medical Center Utrecht, Utrecht, The Netherlands; Department of Radiation Sciences, University of Umeå, Umeå, Sweden; Department of Surgical and Perioperative Sciences, Umeå University, Umeå, Sweden; Department of Community Medicine, Faculty of Health Sciences, University of Tromsø, Tromsø, Norway; Department of Research, Cancer Registry of Norway, Oslo, Norway; Department of Medical Epidemiology and Biostatistics, Karolinska Institutet, Stockholm, Sweden; Samfundet Folkhälsan, Helsinki, Finland; School of Clinical Medicine, University of Cambridge, Cambridge, UK; Cancer Epidemiology Unit, Nuffield Department of Population Health, University of Oxford, Oxford, UK; Division of Clinical Epidemiology and Aging Research, German Cancer Research Center (DKFZ) Heidelberg, Im Neuenheimer Feld 581, D-69120 Heidelberg, Germany

## Abstract

**Introduction:**

The relationship between circulating prolactin and invasive breast cancer has been investigated previously, but the association between prolactin levels and *in situ* breast cancer risk has received less attention.

**Methods:**

We analysed the relationship between pre-diagnostic prolactin levels and the risk of *in situ* breast cancer overall, and by menopausal status and use of postmenopausal hormone therapy (HT) at blood donation. Conditional logistic regression was used to assess this association in a case-control study nested within the European Prospective Investigation into Cancer and Nutrition (EPIC) cohort, including 307 *in situ* breast cancer cases and their matched control subjects.

**Results:**

We found a significant positive association between higher circulating prolactin levels and risk of *in situ* breast cancer among all women [pre-and postmenopausal combined, OR_log2_ = 1.35 (95% CI 1.04-1.76), P_trend_ = 0.03]. No statistically significant heterogeneity was found between prolactin levels and *in situ* cancer risk by menopausal status (P_het_ = 0.98) or baseline HT use (P_het_ = 0.20), although the observed association was more pronounced among postmenopausal women using HT compared to non-users (P_trend_ = 0.06 *vs* P_trend_ = 0.35). In subgroup analyses, the observed positive association was strongest in women diagnosed with *in situ* breast tumors <4 years compared to ≥4 years after blood donation (P_trend_ = 0.01 *vs* P_trend_ = 0.63; P_het_ = 0.04) and among nulliparous women compared to parous women (P_trend_ = 0.03 *vs* P_trend_ = 0.15; P_het_ = 0.07).

**Conclusions:**

Our data extends prior research linking prolactin and invasive breast cancer to the outcome of *in situ* breast tumours and shows that higher circulating prolactin is associated with increased risk of *in situ* breast cancer.

## Introduction

*In situ* breast tumours account for about 20% of all breast cancers diagnosed by mammography [1] and approximately a third of invasive breast cancers are reported to originate from breast carcinoma *in situ* [1-3], which may be a precursor of invasive breast cancer [4]. The focus on *in situ* breast cancer, therefore, offers the advantage of exploring the associations with risk factors that are important early in the carcinogenic process (that is, prior to development of invasive disease) and early in the natural history of breast cancer, increasing our understanding of the aetiology of breast cancer. Identifying common versus distinct risk factors for *in situ* and invasive disease will, in part, provide insight into the common versus distinct mechanisms through which these cancers develop. Further, *in situ* breast cancers are relatively aggressively treated with surgery, radiation and/or hormone therapies [1], underscoring the importance of identifying risk factors for this breast cancer subtype.

The relationship between circulating prolactin and invasive breast cancer risk has been investigated previously [5,6]. In our previous study we found a modest positive association between circulating prolactin levels and invasive breast cancer risk among postmenopausal women (odds ratio (OR)_Q4-Q1_ = 1.29 (95% CI 1.05,1.58), *P*_trend_ = 0.09) [5].

The association between prolactin and *in situ* breast cancer risk, however, has received less attention. In the Nurses’ Health Study, the only large-scale prospective study reporting estimates separately for women with invasive and *in situ* lesions, the increased risk appeared to be confined primarily to invasive cancers (OR_Q4-Q1_ = 1.38 (95% CI 1.11, 1.73), *P*_trend_ = 0.0005 for invasive versus OR_Q4-Q1_ = 1.16 (95% CI 0.77, 1.74), *P*_trend_ = 0.23 for *in situ* breast cancer among postmenopausal women], although there was no heterogeneity comparing *in situ* versus invasive cancer (*P*-value for heterogeneity (*P*_het_) = 0.81) [6]. The only other study to date that included subjects with *in situ* breast cancer did not provide estimates separately by *in situ* versus invasive disease [7].

Therefore, in this study we examined the association between pre-diagnostic prolactin concentrations among pre- and postmenopausal women with subsequent risk of *in situ* breast cancer overall, by menopausal status and by use of postmenopausal hormone therapy (HT) at the time of blood donation within the European Prospective Investigation into Cancer and Nutrition (EPIC) cohort.

## Methods

### The EPIC cohort

The EPIC cohort is based on 366,521 women and 153,457 men recruited between the years 1992 and 2000 in 10 European countries: Denmark, France, Germany, Greece, Italy, Norway, Spain, Sweden, The Netherlands, and the United Kingdom. Details on the subject recruitment, baseline data and blood collection protocols have been reported previously [8]. All EPIC study participants provided written consent for the use of questionnaire information and blood samples in research studies. Ethical approval for the EPIC study was obtained from the ethical review boards of the International Agency for Research on Cancer (IARC) (Lyon, France) and from the local ethics committees in participating countries/study centres (the names of all local ethics committees that approved the study are available Acknowledgements). The current study was additionally approved by the ethical review board at IARC (which holds the central biorepository for EPIC) and the ethical review board in Heidelberg (Heidelberg University), where the laboratory assays and statistical analyses were performed.

Details on vital-status follow up and ascertainment of breast cancer incidence across the study centres were reported previously [9]. Briefly, in all countries (except for France, Greece, and Germany) incident breast cancer cases were identified using a combination of methods employing record linkage with cancer and pathology registries. In Greece, Germany, and France, active follow up of cancer was through health insurance records and direct contact with participants. Self-reported breast cancer cases were all systematically verified from clinical and pathologic records. Breast cancer *in situ* incidence data were coded according to the International Classification of Disease (ICD-10 code D05). For the present study, the closure date for follow up was the last date of complete follow up for both cancer incidence and vital status, which ranged from 2003 to 2006, depending on the study centre. Sweden was not included in the analysis because independent studies were being completed on breast cancer risk and endogenous hormones [7].

### Design of the nested case-control study

General criteria for the inclusion of cases and matched controls into the study were: (1) women had an available blood sample; (2) could be clearly classified as being either premenopausal or postmenopausal at the time they provided their blood sample, and (3) did not have any previous diagnosis of cancer (except non-melanoma skin cancer). For each case, one matched control (closest to the case, based on matching criteria) with an available blood sample was chosen using incidence density sampling among appropriate risk sets consisting of all cohort members who were alive and free of cancer at the time of diagnosis of the index case. Matching criteria were: study recruitment centre, menopausal status at blood donation, age at blood donation (±6 months), time at blood donation (±2 h), fasting status, phase of menstrual cycle (matching categories for premenopausal women: early follicular (days 0 to 7 of the cycle), late follicular (days 8 to 11), mid-cycle (days 12 to 16), early luteal (days 17 to 19), mid luteal (days 20 to 24), late luteal (days 25+), and current use of HT at the time of blood donation (for postmenopausal women).

In total, 307 *in situ* breast cancer cases and an equal number of control subjects were selected for the study. In our analysis, the majority of the cases (91%) were defined as ductal carcinoma *in situ* and 9% as lobular carcinoma *in situ* or carcinoma *in situ* NOS (not otherwise specified).

### Laboratory assays

Prolactin assays were performed in the laboratory of the Division of Cancer Epidemiology at the German Cancer Research Centre (DKFZ) and determined by immunoradiometric assay (IRMA (CT), IBL International GMBH, Germany) in blood collected at baseline. The case-control pairs were analysed in the same batch and quality controls were included in each analytical batch. Laboratory personnel were blinded to the case-control status of samples and quality controls. The detection range of the assay was 0.35 to 133 ng/mL. The mean inter- and intra-assay coefficients of variation were 4.62% and 2.17%, respectively.

### Statistical analyses

Conditional logistic regression was used to estimate ORs and 95% CI for breast cancer *in situ* occurrence. ORs were calculated across tertiles of circulating prolactin and on a continuous log_2_ scale. Linear trends for OR estimates were calculated over a continuous log_2_ scale of prolactin. Circulating prolactin levels vary by menopausal status [10], and therefore the tertile cutpoints were defined separately for pre- and postmenopausal women, based on distributions in the control population. We decided a priori to adjust all models for parity (nulliparous, parous, or missing data), smoking status (current, never, previous, missing data) and body mass index (BMI) in kg/m^2^ (continuous scale), due to modest variation in prolactin levels over these factors in healthy women [10]. A more detailed description of the statistical analysis (including other variables tested for possible confounding) used in this study is described previously [5]. Additionally, for the comparison of the results from our present analysis on *in situ* breast cancer and our prior results on invasive breast cancer [5], a case-case analysis was performed [11]. All *P*-values presented are two-sided and *P* <0.05 was considered statistically significant. Statistical analyses were conducted using SAS software, version 9.2 (SAS Institute, Cary, NC, USA).

## Results

This nested case-control study consisted of 307 cases diagnosed with *in situ* breast cancer, and an equal number of controls. For the premenopausal case-control pairs, the median age at blood donation was 46.4 years (range 34.1 to 56.4 years), and the median age at diagnosis for the cases was 50.6 years (range 37.3 to 61.3) (Table 1). Among postmenopausal women the ages at blood donation and diagnosis were 58.1 years (range 47.7 to 71.4) and 62.8 years (range 51.0 to 78.3), respectively. The median follow-up time between blood donation and date of diagnosis was 4.2 years (range 0.02 to 10.7 years). This study included 82 postmenopausal case-control pairs who used HT at the time they provided their baseline blood sample. Out of 307 *in situ* cases selected to this analysis, 15 patients developed invasive breast cancer after diagnosis of *in situ* disease during further follow up. The median levels of prolactin were 8.3 ng/mL (range 3.8 to 28.1 ng/mL) for patients who developed invasive disease after diagnosis of *in situ* disease and 6.8 ng/mL (range 2.6 to 128.3 ng/mL) in patients who did not develop invasive disease during further follow up (data not shown).

**Table 1:** **Baseline characteristics of** ***in situ*** **breast cancer case and control subjects (data presented as median [min, max] or** ***n*** **[%])**

	Premenopausal women*		Postmenopausal women*	
	**Cases (n = 86)**	**Controls (n = 86)**	**Cases (n = 221)**	**Controls (n = 221)**
Age at blood donation, years	46.4 (34.1, 56.4)	46.5 (34.4, 55.6)	58.1 (47.7, 70.6)	58.0 (47.7, 71.4)
Age at menopause, years			50.0 (33.0, 59.0)	50.0(29.0,58.0)
Age at diagnosis, years	50.6 (37.3, 61.3)		62.8 (51.0, 78.3)	
Lag time till diagnosis, years	4.1 (0.02, 10.0)		4.4 (0.02, 10.7)	
Body mass index, kg/m^2^	23.3 (17.1, 32.8)	24.0 (17.4, 37.2)	24.9 (17.8, 40.8)	24.5 (18.2, 45.9)
Ever had a full-term pregnancy	68 (82.9)	70 (81.4)	190 (86.8)	188 (85.5)
Baseline smoking	15 (17.4)	20 (23.3)	25 (11.3)	33 (14.9)
Baseline use of hormone therapy			82 (37.1)	82 (37.1)
Prolactin levels, ng/mL	8.9 (3.4, 51.7)	7.7 (2.9, 133.0)	6.2 (2.6, 128.3)	5.9 (2.6, 55.3)

Data are presented as median (minimum, maximum) or number **(**%). *Menopausal status at the time of blood donation.

In conditional logistic regression analyses adjusting for parity, smoking status, and BMI, there was a positive association between prolactin concentrations and *in situ* breast cancer risk among all women (pre-and postmenopausal combined), with a statistically significant 35% increase in risk for each one unit increase in prolactin on the continuous log_2_ scale (OR_log2_ = 1.35 (95% CI 1.04, 1.76), P_trend_ = 0.03) (Table 2). The association was similar comparing the top to the bottom tertiles of prolactin, but did not reach statistical significance (OR_Q3-Q1_ = 1.37 (95% CI 0.87, 2.16)). We observed no statistically significant heterogeneity in stratified analyses by menopausal status (*P*_het_ = 0.98). However, after additional stratification by baseline HT use among postmenopausal women, we observed a suggestive association among the HT users (OR_log2_ = 1.77 (95% CI 0.98, 3.21), *P*_trend_ = 0.06), but not among the non-users (OR_log2_ = 1.20 (95% CI 0.82, 1.76), P_trend_ = 0.35). However, case numbers in these subgroups were small, CIs overlapping and the test for heterogeneity between HT users and non-users was not statistically significant (*P*_het_ = 0.20).

**Table 2:** **Adjusted odd ratios** ^**a**^ **(OR) for** ***in situ*** **breast cancer by tertile levels and on a continuous log**_**2**_ **scale of circulating prolactin**

		**Tertiles**		**Log**_**2**_		
	**1**	**2**	**3**	**Odds ratio (95% CI)**	***P***_**trend**_^**b**^	***P***_**het**_^**c**^
**All women**						
Ca/Co	89/101	94/104	124/102			
OR	Ref.	0.98 (0.65, 1.49)	1.37 (0.87, 2.16)	1.35 (1.04, 1.76)	0.03	
**Premenopausal**						
Ca/Co	27/29	19/28	40/29			
OR	Ref.	0.63 (0.25, 1.58)	1.49 (0.59, 3.74)	1.30 (0.80, 2.10)	0.28	
**Postmenopausal (all)**						
Ca/Co	65/72	69/75	87/74			0.98
OR	Ref.	1.00 (0.61, 1.63)	1.31 (0.77, 2.22)	1.38 (1.00, 1.91)	0.05	
**Postmenopausal non-HT users**						
Ca/Co	50/51	39/49	50/39			
OR	Ref.	0.84 (0.44, 1.6)	1.27 (0.65, 2.47)	1.20 (0.82, 1.76)	0.35	
**Postmenopausal HT users**						
Ca/Co	15/21	30/26	37/35			0.20
OR	Ref.	1.63 (0.70, 3.81)	1.62 (0.64, 4.09)	1.77 (0.98, 3.21)	0.06	

^a^Adjusted for parity (nulliparous, parous, or missing data), smoking status (current, never, previous, or missing data) and body mass index (continuous scale); ^b^linear trends for odds ratio estimates over a continuous scale of prolactin levels; ^c^statistical tests for heterogeneity were based on the likelihood-ratio test, comparing the model fit for logistic regression models with and without a corresponding interaction term. Ca/Co, Case/Control; Ref., reference; HT, hormone therapy.

In subgroup analyses (Table 3), we observed significant heterogeneity in the strength of the association with *in situ* breast cancer risk by time between blood donation and diagnosis (that is, lag time) among all women (*P*_het_ = 0.04). Higher concentrations of prolactin were significantly associated with *in situ* breast cancer diagnosed less than 4 years since blood donation (OR_log2_ = 1.78 (95% CI 1.12, 2.84), *P*_trend_ = 0.01), but not with breast cancer diagnosed 4 or more years since blood donation (OR_log2_ = 1.09 (95% CI 0.77, 1.55), *P*_trend_ = 0.63; *P*_het_ = 0.04). Similarly, the estimated association of prolactin with *in situ* breast cancer risk appeared stronger among nulliparous women compared to parous women, more pronounced in analysis restricted to postmenopausal women (OR_log2_ = 5.10 (95% CI 1.23, 21.15), *P*_trend_ = 0.02 in nulliparous women versus OR_log2_ = 1.22 (95% CI 0.88, 1.68), *P*_trend_ = 0.24 in parous women; *P*_het_ = 0.02). It should be noted, however, that the case numbers for nulliparous women were small and the CIs for risk estimates wide. There was no significant heterogeneity by age at tumour diagnosis in either premenopausal or postmenopausal women (*P*_het_ = 0.87) (data not shown).

**Table 3:** **Adjusted odds ratios** ^**a**^ **(OR) for** ***in situ*** **breast cancer on a continuous log**_**2**_ **scale of circulating prolactin by subgroup analysis**

	**Case-control sets, number**	**Log**_**2**_ **odds ratio (95% CI)**	***P***_**trend**_^**b**^	***P***_**het**_^**c**^
**All women**				
Lag time till diagnosis				
<4 years	127	1.78 (1.12, 2.84)	0.01	0.04
≥4 years	180	1.09 (0.77, 1.55)	0.63	
Parity^d^				
Nulliparous	42/45	2.64 (1.07, 6.51)	0.03	0.07
Parous	251/251	1.21 (0.94, 1.57)	0.15	
**Premenopausal women**				
Lag time till diagnosis				
<4 years	39	2.30 (0.85, 6.14)	0.10	0.15
≥4 years	47	0.97 (0.51, 1.83)	0.92	
**Postmenopausal women (all)**				
Lag time till diagnosis				
<4 years	88	1.66 (0.97, 2.85)	0.07	0.19
≥4 years	133	1.18 (0.76, 1.82)	0.46	
Parity^d^				
Nulliparous	29/32	5.10 (1.23, 21.15)	0.02	0.02
Parous	187/185	1.22 (0.88, 1.68)	0.24	
**Postmenopausal non-HT users**				
Lag time till diagnosis				
<4 years	52	1.20 (0.70, 2.05)	0.51	0.52
≥4 years	87	1.08 (0.61, 1.93)	0.79	
**Postmenopausal HT users**				
Lag time till diagnosis				
<4 years	36	6.02 (1.31, 27.72)	0.02	0.04
≥4 years	46	1.29 (0.64, 2.61)	0.47	

^a^Adjusted for parity (nulliparous, parous, missing data), smoking status (current, never, previous, or missing data) and body mass index (continuous scale); ^b^linear trends for odds ratio estimates over a continuous scale of prolactin levels; ^c^heterogeneity between subgroups was based on the chi-square test; ^d^using unconditional logistic regression, adjusted for age and time at blood donation, fasting and smoking status, body mass index, phase of menstrual cycle for premenopausal women and current use of hormone therapy (HT) for postmenopausal women.

Additional statistical analyses showed no evidence for major confounding effects by other lifestyle and reproductive factors nor was there an interaction between prolactin and BMI (*P*_interaction_ = 0.84) (data not shown).

Finally, we compared the results from our present analysis on *in situ* breast cancer and from our prior analysis on invasive breast cancer [5]. The median levels of prolactin comparing *in situ* (n = 307) versus invasive breast cancer tumors (n = 2250) were 8.9 (*in situ*) versus 8.6 ng/mL (invasive) for premenopausal women and 6.2 (*in situ*) versus 6.1 (invasive) ng/mL for the postmenopausal women (data not shown). A heterogeneity test comparing *in situ* versus invasive disease was not statistically significant (*P*_het_ = 0.25 for premenopausal women and *P*_het_ = 0.33 for postmenopausal women).

## Discussion

In this prospective study, we observed a significant positive association between pre-diagnostic circulating prolactin levels and risk of *in situ* breast cancer. Our data showed no evidence for heterogeneity in the relationship of prolactin levels and *in situ* cancer risk by menopausal status or postmenopausal HT use at blood donation, although the observed positive association was more pronounced among HT users versus non-users. In addition, the associations were strongest among women diagnosed with *in situ* breast tumors less than 4 years after blood donation and also among nulliparous women.

Our findings of modest, positive associations between prolactin and *in situ* breast cancer risk are similar to those observed for invasive breast cancer. Prior analyses limited to invasive cases found modest significant associations between circulating prolactin and risk of invasive breast cancer, with up to a 50% increase in risk contrasting top *versus* bottom quartiles [5,6]. Moreover, comparable to our previous findings for invasive breast cancer [5], the observed association in the present study of *in situ* breast cancer was more pronounced among postmenopausal HT users as compared to non-users. Although *in situ* carcinomas in the breast may not have invasive characteristics [12], up to 50% of *in situ* lesions, if left untreated, progress to invasive disease [3]. Little is known about the exact mechanisms influencing the possible progression of *in situ* lesions to invasive disease. However, the very similar associations between prolactin and breast cancer risk observed in our previous study of invasive disease [5] and the current study of *in situ* disease suggest that circulating prolactin may influence risk of invasive and *in situ* breast cancer via the same aetiological pathway. Prolactin may also play a role in progression from *in situ* to invasive breast cancer. However, out of 307 *in situ* cases selected for this analysis, only 15 developed invasive breast cancer during further follow up. Therefore, we were unable to assess the association between prolactin and progression from *in situ* to invasive disease in this study.

Although a vast body of evidence from animal, *in vitro,* and epidemiological studies strongly supports the involvement of prolactin in breast cancer development [5,6,13,14], the complex and diverse biological and molecular mechanisms through which prolactin may increase risk of breast cancer are not clear. Some proposed mechanisms include its proliferative effects on malignant breast cells, mitogenic action, and inhibition of apoptosis via signalling through the prolactin receptor [14]. In addition to the endocrine (circulating) concentrations, locally produced prolactin may promote cancer development via autocrine and paracrine effects [15]. Evidence is also emerging that drugs resulting in elevated prolactin (for example, neuroleptic and hormonal medications) may increase breast cancer risk [16,17].

The relationship between prolactin and *in situ* breast cancer risk was confined to tumors diagnosed within the first 4 years from blood donation. Interestingly, this differential association by time between blood collection and diagnosis was not evident for invasive breast cancer risk in our previous analysis [5]. Whether prolactin is involved in the early development or promotes late-stage growth of an established tumor is unclear. Consistent with the current analysis, recent results from the Nurses’ Health Study suggest that prolactin primarily plays a role in the late stage of tumor development, with stronger positive associations observed among participants providing blood samples closer to diagnosis [6,18]. This is also supported by studies observing elevated levels of circulating prolactin in breast cancer patients [19], and demonstrating the ability of breast tumors to secrete prolactin [13]. However, our present analysis suggests that prolactin may also operate early in the natural history of breast cancer by increasing risk of *in situ* tumors, the earliest detectable breast carcinomas.

In the current study, prolactin was associated with *in situ* breast cancer among postmenopausal nulliparous women but not in parous women. Parity, a well-established protective factor for breast cancer [20], has been associated with a long-term post-pregnancy reduction in levels of circulating prolactin [10,21]. Our results support the hypothesis that lowered prolactin levels following pregnancy might serve as one of several possible mechanisms that mediate the long-term reduction in risk afforded by parity. However, our findings should be interpreted with caution as only about 15% of women in this case-control study were nulliparous and OR estimates for nulliparous women had wide confidence limits.

Our study is the second largest prospective study on the association between prolactin and breast cancer risk, to provide risk estimates for *in situ* breast cancer. A general limitation of our study is that prolactin levels were measured only at a single point in time. However, reliability studies in which repeat blood samples were taken over intervals of up to three years have shown relatively high intra-class correlation (ICC coefficients of up to 0.76) between individuals’ serum prolactin levels over time [22-24]. In *situ* breast cancer is largely diagnosed as a result of mammography; however, we were not able to adjust our models for the participation in mammographic screening, nor were we able to compare rates of screening in cases and controls. Our study had relatively limited numbers of case subjects for subgroup analysis, and thus the statistical power for detecting associations of prolactin with risk in these subgroups was limited. Due to very limited information available for the molecular characteristics of *in situ* tumors, we were not able to perform the analysis by molecular subtypes of the lesions.

## Conclusions

In conclusion, we have extended prior research showing an association between prolactin and invasive breast cancer to the outcome of *in situ* breast cancer. Our study shows that higher circulating levels of prolactin may be associated with increased risk of *in situ* breast cancer. It is estimated that up to 50% of untreated *in situ* breast lesions will progress to invasive disease. However, we are currently not able to distinguish between indolent and more aggressive *in situ* carcinomas, and the side effects of the medical management of *in situ* lesions (for example, lumpectomy, radiation and/or hormone therapy) are not negligible. Therefore, identification of risk factors for *in situ* breast carcinomas is important both from the perspective of characterizing the risk of the disease as diagnosed and treated, as well as providing insight into the aetiology of the *in situ* carcinomas, which represent pre-invasive disease. Additional work to characterize circulating prolactin and risk of invasive disease, particularly after the development of *in situ* breast disease, is needed.

## Abbreviations

BMI: body mass index
EPIC: European Prospective Investigation into Cancer and Nutrition
HT: hormone therapy
IARC: International Agency for Research on Cancer
ICC: intra-class correlation
ICD: International Classification of Diseases for Oncology
OR: odds ratio
Q4-Q1: Quartile4-Quartile1
